# Additional value of associating aortic valve calcification to coronary calcium as a gatekeeper for coronary tomography angiography

**DOI:** 10.1186/s12872-015-0058-5

**Published:** 2015-07-01

**Authors:** Ana Faustino, Rui Providência, Luís Paiva, Rui Catarino, Susana Basso, Marco Costa, Lino Gonçalves

**Affiliations:** Cardiology Department, Coimbra’s Hospital and University Centre – General Hospital, 3041-801 S. Martinho do Bispo, Coimbra, Portugal; Faculty of Medicine, University of Coimbra, Coimbra, Portugal; Radiology Department, Coimbra’s Hospital and University Centre – General Hospital, Coimbra, Portugal

**Keywords:** Aortic valve calcification, Cardiac computed tomography, Coronary artery calcification, Coronary artery disease, Coronary CT angiography

## Abstract

**Background:**

Aortic valve calcification shares risk factors with coronary artery disease. Coronary calcium has been used has a gatekeeper to performing coronary tomography angiography. The aim of this study was to evaluate aortic valve calcification as a predictor of obstructive coronary artery disease by computed tomography, and its possible usefulness, alongside with coronary calcium, to improve the decision of whether or not to proceed with computed tomography angiography.

**Methods:**

Transversal case–control study including 154 consecutive patients (62 ± 12 years, 57.6 % female, without known coronary or valve disease) undergoing calcium scoring and angiography through computed tomography (Phillips Brilliance, 16-slice). Predictors of aortic valve calcification and obstructive coronary artery disease were identified. Usefulness of aortic valve calcification when added to calcium score for prediction of obstructive coronary artery disease was assessed by binary logistic regression and net reclassification index.

**Results:**

Aortic valve calcification was associated with higher coronary calcium, extent and prevalence of obstructive coronary disease, which was identified in 22.1 % of patients and was discriminated by aortic valve calcium with an area under curve 0.749 (*p* < 0.001, Youden index: 61). A higher discriminative power was achieved with a model based on coronary and aortic valve calcification (AUC 0.900, *p* < 0.001). Compared with calcium score >400 as a gatekeeper to angiography, the association of aortic calcium >61 allowed a net reclassification index of +7.7 % of patients.

**Conclusions:**

Aortic valve calcification is associated with the prevalence and extent of obstructive coronary artery disease by computed tomography angiography and is an easy, fast and useful method to improve the selection of patients for angiography.

## Background

Coronary artery calcium (CAC) can be assessed by using non-contrast cardiac computed tomography, with low radiation exposure, and is usually assessed through the “Agatston score”. This is a marker of coronary atherosclerosis and reflects the total atherosclerotic burden, including calcified and non-calcified plaques [1].

CAC has been extensively validated as a marker of cardiovascular risk, with values greater than or equal to 400 allowing reclassification of low/intermediate-risk asymptomatic patients into high risk [2].

CAC also has also been associated with coronary luminal stenosis, with high specificity (>72 % %) for values over 310 – 400 [3, 4]. Due to this surrogate value of CAC to predict coronary stenosis, its use has been suggested as a gatekeeper to the use of cardiac computed tomography (CT) angiography (CTA) for diagnosing significant coronary artery disease (CAD) in patients with chest pain [5]. The appropriate use criteria for CT published in 2010 considered inappropriate to perform a CTA for CAC score values higher than 400 [6]. Guidelines from the National Institute of Health and Clinical Excellence also recommend opting for CTA in CAC scores of 1–400 and choosing invasive angiography in CAC scores above 400 [7].

Aortic valve calcification (AoVC) shares some risk factors with CAD [8] and is believed to be associated with cardiovascular risk [9, 10], coronary plaque burden [11, 12] and severity of angiographic coronary artery disease [13]. However, its role as a predictor of obstructive CAD identified by CTA and its possible usefulness, on top of the calcium score, is unknown. The reproducibility of AoVC measurement by CT has been previously reported [14], and can be reliably measured using the “Agatston score” in the same acquisition of CAC, without contrast or additional radiation exposure [8–14].

This study aims to evaluate AoVC, identified by CTA, as a predictor of obstructive CAD and the usefulness of combining AoVC and CAC score to decide whether or not to proceed with CTA.

## Methods

### Study design

Transversal case–control single centre study including patients referred for assessment of possible CAD using CT. CAC and CTA were performed. Clinical and laboratorial data were collected. This study was approved by our Institution’s Cardiology Department Supervisor and Ethics Committee in December 2008. All patients provided informed consent before undergoing CT.

### Patients and eligibility criteria

A total of 154 consecutive patients, without known history of severe aortic stenosis or coronary artery disease, were referred for CT for evaluation of coronary artery disease, in a non-acute setting, between January 8, 2009, and September 30, 2010. Patients over 18 years old were included in the study. Exclusion criteria were contraindication to iodine-based contrast agents, glomerular filtration rate <30 mL/min, pregnancy, inability to sustain a 15-s breath-hold, cardiac arrhythmias or uninterpretable CTA.

### Initial data collection

An extensive review of clinical records from our outpatient clinic, hospital ward and emergency department admission(s), was performed by 2 co-investigators, and the following data were collected: demographic features, cardiovascular risk factors, cardiovascular risk scores (SCORE [15] and Framingham [16]), previous medical history (including history of CAD and severe aortic stenosis), physical examination (including weight, height, body mass index, blood pressure and heart rate) and analytical study: total cholesterol, triglyceride, high density lipoprotein cholesterol, low density lipoprotein cholesterol, serum creatinine and C-reactive protein. GFR was calculated by MDRD (Modification of Diet in Renal Disease) formula.

Hypertension, dyslipidemia and diabetes were defined by self-reporting during history-taking and/or history or use of specific therapy. Family history of CAD was defined as fatal or non-fatal myocardial infarction or coronary revascularisation in first-degree male relatives <55 years or first degree female relatives <65 years. History of cigarette smoking was considered present if a subject was a current or former smoker.

### CT data acquisition

All scans were performed using a 16-slice CT scanner (Brilliance 16; Philips Medical Systems, Eindhoven, the Netherlands). A prospective scan without contrast enhancement was performed to measure CAC and AoVC (sequential scan with 8 × 3 mm collimation, tube current 55mAs at 120 kV, 3 mm width), followed by 16-slice contrast-enhanced spiral scan of the heart performed with ECG gating and retrospective post processing. CTA parameters: 16 × 0.75 mm collimation, 400 ms gantry rotation, pitch of 0.298, tube voltage at 120 kV, maximum current of 600—800 mAs depending on patient size, half-scan reconstruction mode and imaging craniocaudal direction. All patients received 5 mg of sublingual isosorbide dinitrate 5 min before CTA acquisition. Patients with a heart rate >65 bpm received 50-200 mg of oral metoprolol. A bolus (120-130 mL) of iodinated contrast agent (370 mOsm) was intravenously injected (4–5.5 ml/s). A region of interest was placed in the descending thoracic aorta and image acquisition was automatically initiated using bolus tracking (selected threshold: 110 Hounsfield units [HU]). Images were reconstructed in five phases of the cardiac cycle (0, 37.5, 62.5, 75 and 87.5 % of the R-R interval) to minimize motion artifacts. The estimated effective radiation dose used was: per CAC and AoVC - median 0,8 mSv (maximum: 0,32 mSv; minimum: 1.28 mSv); per CTA – median 10,44 mSv, maximum 17,44 mSv, minimum 9,24 mSv (CTA per se: median 10 mSv, maximum 17 mSv, minimum 8,8 mSv; Locator: median 0,44 mSv, maximum 1,28 mSv, minimum 0,32 mSv); per complete scan: median 11 mSv (maximum 18 mSv, minimum 10 mSv).

### CT Image interpretation

CTA image evaluation was performed on a separate 3D workstation (Brilliance workstation, Philips Medical Systems, Eindhoven, the Netherlands) by two experienced reviewers. CTA were analysed by assessment of axial slices, multiplanar reformations (along the vessel axis and cross-sectional images), and the three thin-slab maximum intensity projections. The coronary artery tree was divided into proximal, medial and distal, according to classic angiographic definition. Plaques were classified as obstructive or non-obstructive using a 50 % threshold of luminal narrowing. The presence of obstructive CAD was defined by >50 % lumen narrowing, and was classified according to the number of vessels with obstructive CAD: single-vessel disease (one vessel) or multivessel disease (two or three vessels). Plaques were defined as structures >1 mm^2^ within and/or adjacent to the vessel lumen, distinct from lumen and surrounding tissue. Plaques were classified as: calcified – if they had more than 50 % calcified tissue (density >130HU in native scans), mixed – if composed with <50 % calcium, and non-calcified lesions - without any calcium. After independent assessment, the final diagnosis was obtained by a consensus interpretation of the two reviewers.

CAC and AoVC were measured using a workstation (Aquarius iNtuition, version 4.4, Tera Recon, Inc, San Mateo, CA, USA). CAC was measured for each patient using the automatic calcium detection algorithm of the workstation, according to Agatston method, with a calcium threshold of 130 HU. AoVC was measured and quantified with the same lesion definition, the same software and the same acquisition of CAC. The presence of AoVC was defined as any calcified lesion detected within the aortic valve leaflet area or extending to the aortic root. Calcium within the aortic sinuses or thoracic aorta was excluded from analysis and not measured as AoVC.

### Study endpoints

The primary endpoint of this study was detection of obstructive CAD by CTA.

The secondary endpoint was the identification of patients with CAC <400 and AoVC >61 or AoVC >454 with obstructive CAD.

### Statistical analysis

Statistical analysis was performed using SPSS, v. 17.0. Baseline characteristics were described with counts and proportions for categorical data; continuous variables with normal distribution were described by mean ± standard deviation, while continuous variables with non-normal distribution were described with median, minimum and maximum value. The Kolmogorov-Smirnov test was used to test the normal distribution of continuous variables. The Chi-square test, Student’s *t*-test and non-parametric equivalent tests were used when appropriate. Regression estimation techniques were applied to replace missing values whenever the number of missing values was negligible, otherwise cases with missing values would be omitted. *P* values <0.05 (two-sided) were considered statistically significant.

A comparative analysis was performed to evaluate potential predictors of AoVC. Three cutoffs were used for this purpose: AoVC ≥ 1 (presence of any aortic valve calcification), AoVC > 61 (threshold defined according to the Youden index on ROC curve analysis - optimal sensitivity and specificity: 66.7 % sensitivity and 76.7 % specificity) and AoVC > 454 (cutoff of AoVC with equivalent specificity (95.8 %) to that of CAC > 400 for obstructive CAD in our sample). We also performed a comparative analysis to evaluate a potential association with obstructive CAD. Potential predictors presented as continuous variables were converted into binary variables using as cutoff point the Youden index. Binary logistic regression analysis was performed to identify, among potential associations, the independent predictors of AoVC and obstructive CAD. A predictor model of obstructive CAD was created using CAC and AoVC (PM CAC + AoVC) using continuous variables in binary logistic regression with the method Enter; regression coefficients obtained were then applied to calculate predicted risks according to predictor model. The discriminatory power for predicting obstructive CAD using AoVC, CAC, Framingham, SCORE and the predictor model was then evaluated through receiver operating characteristic (ROC) curves. Comparisons of areas under ROC curves (AUC) were performed using MedCalc for Windows version 9.2.0.1.

Finally, the net reclassification improvement (NRI) was calculated according to the method described by Pencina et al. [17] to quantify the reclassification with CAC > 400 or AOVC > 61/ or >454, comparatively with CAC > 400, as a gatekeeper for CTA. A positive and significant NRI translates a net overall successful reclassification of subjects into more appropriate risk categories (e.g. a patient who reaches the primary endpoint that is reclassified into higher risk groups with CAC and AoVC or a subject who does not reach the primary endpoint that is reclassified into a lower risk category with CAC and AoVC). The amount of overall reclassification is translated by the extent of the NRI (a per cent value). A positive NRI value represents an adequate reclassification into the correct risk category, whereas a negative NRI represents a worse reclassification with the new risk stratification scheme.

## Results

### Study population

One hundred and fifty-four patients were referred for CT. Demographic, clinical and laboratorial characteristics of study population are summarized in Table 1.

**Table 1:** Study population baseline characteristics

Patient baseline characteristics	
Age (years: mean ± sd)	62 ± 12
Female, % (n)	57.6 % (88)
Caucasian, % (n)	100 % (154)
Cigarette smoking, % (n)	14.9 % (23)
Hypertension, % (n)	83.8 % (129)
Dyslipidemia, % (n)	63.6 % (98)
Family history of CAD, % (n)	3.9 % (6)
Body mass index (Kg/m^2^_,_ mean ± sd)	28.7 ± 4.2
Framingham (median [minimum; maximum])	10 [1; 54]
SCORE (median [minimum; maximum])	2 [0; 16]
Glomerular filtration rate (mL/min/1.73 m^2^, mean ± sd)	86.7 ± 22.7
Total cholesterol (mmol/L, mean ± sd)	5.2 ± 1.2
LDL cholesterol (mmol/L, mean ± sd)	3.0 ± 0.9
HDL cholesterol (mmol/L, mean ± sd)	1.3 ± 0.5
Triglycerides (mmol/L, mean ± sd)	1.0 ± 0.9
C-reactive protein (mg/dL: median [minimum; maximum])	0.6 [0.1; 2.1]

### CT results

The median CAC was 20.6 (0; 5515.6) and median AoVC was 9.1 (0; 3667.5). A CAC score of zero was observed in 59 patients (38.3 %) and 54 patients (35.1 %) had an AoVC of zero. Obstructive CAD was identified by CTA in 34 patients (22.1 %).

### Predictors of AoVC

On univariate analysis, for the different cutoffs of AoVC, patients with higher levels of AoVC were older, had higher pulse pressure, higher cardiovascular risk by SCORE, higher prevalence of calcified plaques, of obstructive CAD and number of vessels with obstructive CAD (Table 2). Higher values of CAC were also associated to higher values of AoVC for all assessed cutoffs (Table 2). A higher prevalence of atherosclerosis was observed in patients with AoVC values ≥1 and >61, but not in those with AoVC >454 (Table 2). On multivariate analysis, age was an independent predictor of AoVC for all the cutoffs, while pulse pressure was independent predictor of any calcification and of AoVC >454. Obstructive CAD predicted AoVC > 61 (Table 3).

**Table 2:** Predictors of Aortic Valve calcification on univariate analysis

Predictors of AoVC: univariate analysis	AoVC ≥1			AoVC >61			AoVC >454		
	AoVC ≥1	AoVC =0	p	AoVC >61	AoVC ≤61	p	AoVC >450	AoVC ≤454	p
Male gender	44 %	40.7 %	0.697	36 %	46.2 %	0.233	38.5 %	43.3 %	0.738
Dyslipidemia	70.4 %	84.9 %	0.054	69.4 %	62.7 %	0.423	75 %	64 %	0.445
Diabetes	26.5 %	18.9 %	0.292	22.4 %	24.5 %	0.781	16.7 %	24.5 %	0.543
Smoking	12.2 %	20.8 %	0.165	8.2 %	18.6 %	0.094	8.3 %	15.8 %	0.488
Family history	3.2 %	5.9 %	0.437	0 %	6.3 %	0.074	0 %	4.5 %	0.452
SBP (mmHg)	149 ± 23	137 ± 23	0.003	148 ± 24	143 ± 23	0.248	155 ± 23	144 ± 24	0.105
DBP (mmHg)	76 ± 13	80 ± 12	0.061	75 ± 12	78 ± 13	0.111	75 ± 14	77 ± 13	0.499
PP (mmHg)	73 ± 22	58 ± 19	<0.001	73 ± 21	65 ± 23	0.032	80 ± 26	67 ± 22	0.036
Age (years)	66 ± 10	54 ± 12	<0.001	71 ± 8	58 ± 12	<0.001	74 ± 6	61 ± 12	<0.001
Framingham	15 ± 11	11 ± 11	0.003	14 ± 11	13 ± 11	0.231	17 ± 12	13 ± 11	0.127
SCORE	3.1 ± 2.6	2.0 ± 2.2	0.001	3.2 ± 2.0	2.5 ± 2.7	<0.001	4.1 ± 2.0	2.6 ± 2.6	0.002
GFR (mL/min/1.73 m^2^)	86 ± 24	88 ± 20	0.560	83 ± 21	89 ± 23	0.131	78 ± 18	83 ± 23	0.176
CAC	302 ± 745	96 ± 233	<0.001	490 ± 994	102 ± 229	<0.001	1014 ± 1571	156 ± 383	0.001
Calcified plaque	72.3 %	40.7 %	<0.001	82 %	51.5 %	<0.001	92.3 %	58.6 %	0.017
Coronary Ather.	72.7 %	40.7 %	<0.001	72.7 %	40.7 %	<0.001	69.2 %	47.9 %	0.140
Obstructive CAD	29 %	9.3 %	0.005	44 %	11.5 %	<0.001	53.8 %	19.1 %	0.004
Number of vessels with obstructive CAD	0: 94.4 %	0: 78 %	0.031	0: 68 %	0: 91.3 %	0.001	0: 61.5 %	0: 85.8 %	0.007
	1: 3.7 %	1: 155		1: 22 %	1: 5.8 %		1: 15.4 %	1: 10.6 %	
	>1: 1.9 %	>1: 7 %		>1: 10 %	>1: 2.9 %		>1: 23.1 %	>1: 3.5 %	

AoVC aortic valve calcification; Ather atheroslcerosis; CAC coronary artery calcium; CAD coronary artery disease; DBP diastolic blood pressure; Family history Family history of coronary artery disease; GFR glomerular filtration rate; p significance level; PP pulse pressure; SBP systolic blood pressure

**Table 3:** Predictors of Aortic Valve calcification on multivariate analysis

Predictors of AoVC: multivariate analysis	AoVC ≥1			AoVC >61			AoVC >454		
	OR	95 % CI	p	OR	95 % CI	p	OR	95 % CI	p
Age	1.100	1.057-1.146	<0.001	1.183	1.09-1.261	<0.001	1.209	1.079-1.355	0.001
CAD	------	------	------	4.859	1.694-13.938	0.003	------	------	------
Pulse pressure	1.027	1.007-1.048	0.008	------	------	------	1.039	1.003	1.077

AoVC aortic valve calcification; CAD coronary artery disease; p – significance level

#### AoVC as predictor of obstructive CAD

On univariate analysis, obstructive CAD was associated with male gender, dyslipidemia, age > 64 years, SCORE ≥ 3, CAC >400, AoVC ≥1, AoVC >61 and AoVC >454 (Table 4). Using a general linear model, AoVC remained associated with obstructive CAD after adjustment for age: effect size 0.392, *p* > 0.001. Considering all these variables on multivariate analysis, age and CAC were independent predictors of obstructive CAD (Table 5).

**Table 4:** Predictors of obstructive coronary artery disease on univariate analysis

Predictors of obstructive CAD: univariate analysis	Without Obstructive CAD	With Obstructive CAD	p
Male gender, n% (n)	38.3 % (46)	58.8 % (20)	0.033
Dyslipidemia, % (n)	60.7 % (71)	79.4 % (27)	0.044
Hypertension	84.7 %(100)	85.3 % (29)	0.937
Diabetes	24.8 % (29)	20.6 % (7)	0.613
Family history of CAD	3.6 %(4)	6.1 % (2)	0.528
Cigarette smoking	17.6 % (6)	14.5 % (17)	0.656
Age >64	45.5 % (55)	73.5 % (25)	0.004
SCORE ≥3	28.2 % (33)	61.8 % (21)	<0.001
Framingham ≥11	45.3 % (53)	61.8 % (21)	0.091
CAC ≥400	3.3 % (4)	50 % (17)	<0.001
AoVC ≥1	59.2 % (71)	85.3 % (29)	0.005
AoVC >61	23.3 % (28)	64.7 % (22)	<0.001
AoVC >454	5 % (6)	20.6 % (7)	0.004

AoVC aortic valve calcification; CAC coronary artery calcium; CAD coronary artery disease; p – significance level

**Table 5:** Predictors of obstructive coronary artery disease on multivariate analysis

Predictors of Obstructive CAD: multivariate analysis	OR	95 % CI	p
Age	1.066	1.013-1.122	0.014
CAC	1.005	1.003-1.007	<0.001

CAC coronary artery calcium; CAD coronary artery disease; CI confidence interval; p – level of significance

### AoVC on top of CAC for the decision of whether or not to proceed with CTA

**Fig. 1:**
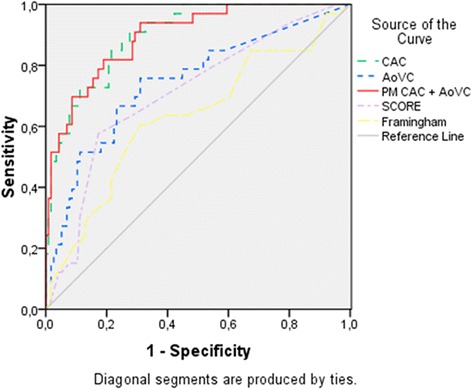
ROC curves for the discrimination of obstructive coronary artery disease: AoVC – AUC 0.749, 95%CI 0.837-0.955, *p* < 0.001; CAC – AUC 0.896, 95%CI 0.837-0.955, *p* < 0.001; PM CAC + AoVC – AUC 0.900, 95%CI 0.838-0.962, *p* < 0.001; SCORE – AUC 0.690, 95%CI 0.577-0.802, *p* < 0.001; Framingham 0.596, 95%CI 0.471-0.721, *p* = 0.120. Legend: AoVC - aortic valve calcification; CAC - coronary artery calcium; CAD - coronary artery disease; PM CAC + AoVC: predictor model of obstructive CAD based on coronary artery calcium and aortic valve calcification

The predictive model of obstructive CAD based on CAC and AoVC had a good calibration (Hosmer and Lemeshow test: *p* = 0.155). On ROC curve analysis, the predictor model showed a good discriminatory power for obstructive CAD, numerically higher than isolated CAC or AoVC (Fig. 1), but without reaching statistical significance, with a difference between AUC of 0.004, 95 % CI: −0.0170 to 0.0258, *p* = 0.684.

The secondary endpoint was reached in 5.9 % (2 of 34) of patients with AoVC >454, and in 29.4 % (10 of 34) of patients with AoVC >61.

The NRI of CAC >400 or AoVC >454 (vs isolated CAC > 400) to not perform CTA was 2.5 %: 5.9 % (2 out of 34) more patients with obstructive CAD would not undergo CTA while 3.3 % (4 out of 120) more patients without obstructive CAD would be spared CTA. Running the same test using the threshold AoVC >61 led to an NRI of 7.7 %, where 29.4 % (10 out of 34) more patients with obstructive CAD and 21.6 % (26 out of 120) more patients without obstructive CAD would not perform CTA.

## Discussion

These data have shown that AoVC is associated with obstructive CAD and CAC. We observed that AoVC can be used to improve the reclassification of high CAD probability in patients with AoVC >61. Indeed, AoVC may be used to refine CAC, providing support to the decision of not to proceed with CTA, thus preventing unnecessary exposure to radiation and iodinated contrast resulting from CTA, that would be futile and inappropriate in patients with obstructive CAD.

This report is innovative because it assesses the usefulness of AoVC as a gatekeeper for CTA, on top of CAC. On ROC curve analysis, the predictor model created by association of AoVC to CAC showed higher discriminatory power than CAC alone, although this difference was not statistically significant. As ROC curve analysis may lack sensitivity, other methods have been proposed for assessing and comparing the accuracy of different risk classifications. Net reclassification improvement index evaluates whether a new model provides a more accurate stratification into higher or lower risk categories of clinical importance [17]. The treshold of AoVC 61 allowed a better reclassification of 7.7 % of patients, which as practical relevance once determination of AoVC is easy, fast and can be performed in the same acquisition of CAC, without increasing risks or costs for each patient. Indeed, it would avoid unnecessary exposure to radiation and iodinated contrast, and would reduce costs related to CTA in 29.4 % of patients with CAD.

The exposure to ionizing radiation is a matter of concern. The effective dose of radiation used in this study was similar to that reported by other groups [18–21]. CAC (and AoVC) was performed with a relatively low radiation exposure (0,8 mSv), whereas CTA increased the exposure to radiation in about 10,44 mSv, what could have been avoided in 29.4 % of patients with CAD, by using the AoVC. Moreover, these patients will possibly perform invasive angiography, being exposed to further 3-5 mSv [20, 22].

The volume of iodinated contrast medium used was also similar to that reported by other groups [21–24]. It is related to costs [25] and possible complications, and could be avoided in some patients by using AoVC.

Still, CTA is significantly more expensive than CAC (costs per study in Portugal: isolated CAC 80€; complete study with CTA 207,1€) [25], and the use of AoVC could reduce unnecessary costs in 29.4 % of patients with CAD. The use of CTA in the acute setting also showed to reduced the costs of care for patients presenting with chest pain and low- or intermediate-risk [26]; for these patients, refining the decision of proceeding with the CTA would be even more important.

In the study population, AoVC was associated to calcified plaques, atherosclerosis, higher levels of CAC, prevalence and extension of obstructive CAD. Sub-studies of ROMICAT (The Rule Out Myocardial Infarction using Computer Assisted Tomography) trial [11] and MESA (The Multi-Ethnic Study of Atherosclerosis) [12] have already demonstrated the relationship of AoVC with the presence and extent of CAC. AoVC was also a marker of the extent and severity of coronary artery disease evaluated by invasive angiography in a sample of 99 patients [13]. Nevertheless, to the best of our knowledge, this is the first report of AoVC as a predictor of obstructive CAD detected by CTA.

AoVC was associated with obstructive CAD in all the evaluated cutoffs and had a good discriminatory power for identifying obstructive CAD, as illustrated by ROC curve analysis.

The AoVC cutoff value = 61 was the threshold with the best amount of sensitivity and specificity for obstructive CAD. The latter was associated with higher levels of AoVC and was an independent predictor of AoVC >61, but this was not verified for the other cutoffs. These data suggest the AoVC cutoff value = 61 as a better indicator of CAD than the value 454, which was equivalent to the threshold of CAC = 400 specificity-wise. The cutoff 61 also achieved a higher net reclassification index, proving to be the most suitable to use as gatekeeper for CTA, on top of CAC.

An association of traditional cardiovascular risk factors, such as hypertension, diabetes, hyperlipidemia, age and male gender, to the presence of AoVC has been previously described [8, 9]. In our sample, age and pulse pressure were associated with AoVC, but no association was found regarding other classical risk factors. They were predictors of higher levels of AoVC, but they were not independent of each other. AoVC was also associated to higher cardiovascular risk defined by SCORE, similarly to what has been observed with other cardiovascular risk classification schemes [9].

Obstructive CAD was also associated with age, and with risk factors as male gender and dyslipidemia.

### Limitations

Our study has several limitations. We describe results of a single-centre study, with a limited number of enrolled patients. A larger sample would better assess the reclassification capabilities of AoVC. Our data were retrospectively collected and evaluated, precluding a better definition of the cardiovascular risk factors evaluated. The definition of these risk factors was based on self-reporting while doing history-taking or in the use of specific therapy, which may have underestimated its true prevalence in this population and could act as a confounding factor for the evaluation of AoVC predictors.

## Conclusions

AoVC is associated with the prevalence and the extent of CAC, and with obstructive CAD on CTA. This association remains even when AoVC is corrected for age. AoVC is an easy and fast method and its use for the improvement of the selection of patients for CTA, on top of CAC values, may justify further evaluation.

## Abbreviations

CAC: Coronary artery calcium
CT: Cardiac computed tomography
CTA: Cardiac computed tomography angiography
CAD: Coronary artery disease
AoVC: Aortic valve calcification
PM CAC + AoVC: Predictor model of obstructive CAD based on coronary artery calcium and aortic valve calcification
